# *TTC21B* variants disrupt the left-right asymmetry and pronephric development in zebrafish

**DOI:** 10.1016/j.gendis.2025.101709

**Published:** 2025-06-06

**Authors:** Linxia Deng, Yuan Yang, Xiaoling Yin, Jing Yang, Yijie Duan, Kang Wang, Weicheng Duan, Yu Zhang, Bo Xiong, Jianhua Zhou

**Affiliations:** a Department of Pediatrics, Tongji Hospital, Tongji Medical College, Huazhong University of Science and Technology, Wuhan, Hubei 430030, China; b Department of Forensic Medicine, Tongji Medical College, Huazhong University of Science and Technology, Wuhan, Hubei 430030, China; c Department of Pediatrics, The Third Xiangya Hospital, Central South University, Changsha, Hunan 410011, China; d Department of Forensic Medicine, Nanjing Medical University, Nanjing, Jiangsu 211166, China

Nephronophthisis (NPHP) is an autosomal recessive kidney disease and is the most prevalent monogenic cause of end-stage renal disease in childhood. The tetratricopeptide repeat domain 21B (*TTC21B*) gene encodes the ciliary protein intraflagellar transport protein 139 (IFT139) and has been recently implicated in heterogeneous diseases, including nephronophthisis type 12 (NPHP12), short-rib thoracic dysplasia 4 (SRTD4), and Joubert syndrome (JBTS).^1^^,^^2^ In Europe and North Africa, the prevalent *TTC21B* variant c.626C > T (p.P209L) has been associated with focal segmental glomerulosclerosis in adults.^2^^,^^3^ To date, only a limited number of *TTC21B* gene variants have been reported in Chinese individuals, predominantly presenting with infantile NPHP, which differs from the manifestations observed in Caucasian patients.^4^^,^^5^ In this study, we identified novel *TTC21B* gene variants in Chinese children with NPHP and investigated their role in left-right asymmetry and pronephric development.

Firstly, we analyzed 5 cases of NPHP12 from 4 non-consanguineous Chinese families, with detailed clinical manifestations summarized in supplementary results and Table S1. Utilizing exome sequencing, we identified 4 novel variants in the *TTC21B* gene: c.1897C > T (p.Q633∗), c.2581C > T (p.Q861∗), c.215_216delGT (p.C72Ffs∗10), and c.262+5G > C (Fig. 1A–D). Surprisingly, 3 out of 4 patients shared the missense variant c.1552T > C (p.C518R), which was also found in 14 other Chinese patients, suggesting that this variant may be a potential hotspot mutation in the Chinese population (Table S1 and Fig. 1A). A comprehensive review of 18 patients carrying the p.C518R variant revealed that the majority presented infantile NPHP and progressed to end-stage renal disease at an average age of 3.5 years (range: 12 days to 16 years). Clinical features included mild to severe proteinuria, hypertension, and extra-renal manifestations such as situs inversus (8/18), congenital heart defects (5/18), cirrhosis or hepatomegaly (4/18), splenomegaly (2/18), and brachydactyly or polydactyly (2/18). These observations suggest that the C518R variant likely substantially impairs the function of the IFT139 protein.

**Figure 1:**
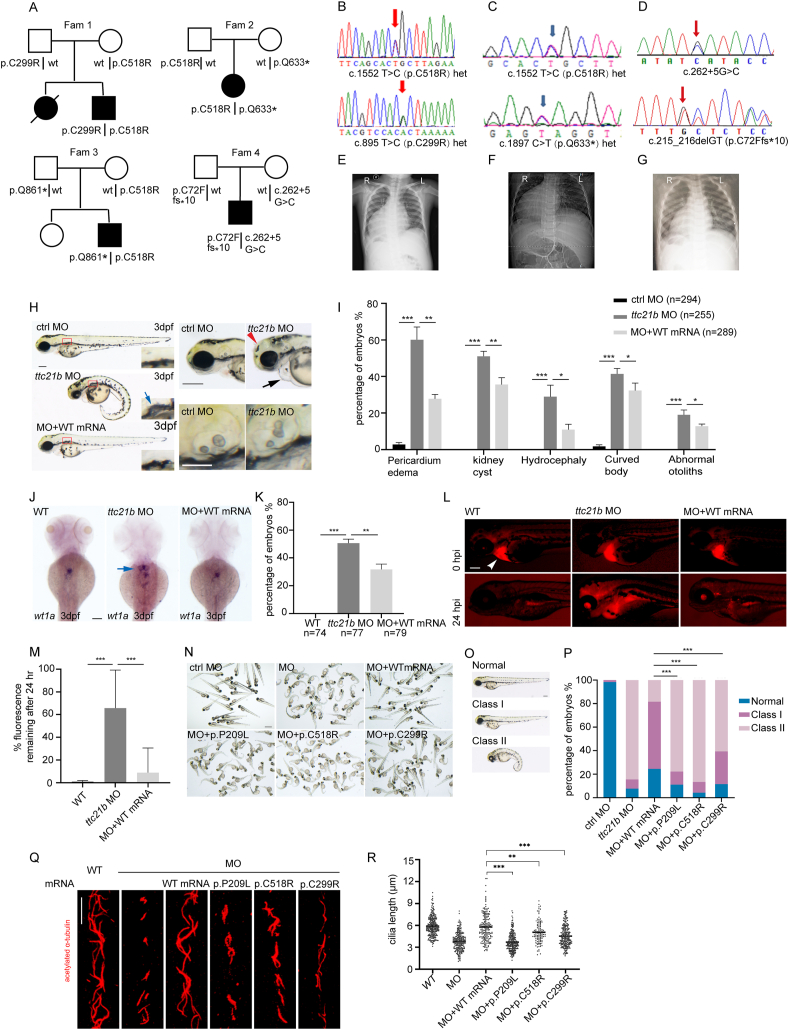
*TTC21B* variants cause nephronophthisis in children and disrupt the cilia and pronephric development in zebrafish. **(A)** Pedigrees of affected families with *TTC21B* variants. **(B**–**D)** Sequence chromatograms in family 1, 2, and 4. **(E**–**G)** The chest X-ray showed situs inversus, where the heart was on the right side. (E) The chest X-ray of the proband's sister of family 1. (F) The chest X-ray of the patient in family 2. (G) The chest X-ray of the patient in family 3. **(H)** Representative images of *ttc21b* morphants at 3 days post-fertilization (dpf) with curved body axis, pronephric cysts (blue arrow; scale bar, 200 μm), hydrocephalus (red arrowhead) and pericardial edema (black arrow; scale bar, 200 μm), and abnormal otoliths in otic vesicles (scale bar, 100 μm). **(I)** Statistical analysis of gross morphology of embryos injected with the indicated morpholinos (MOs) or mRNAs. **(J)** Pronephric cysts validated by whole-mount *in situ* hybridization with the podocyte differentiation marker wt1a. Scale bars, 100 μm. **(K)** Quantification of embryos with pronephric cysts. **(L)** The top and bottom panels indicate the embryos injected into the pericardium with rhodamine dextran at 0 h post-injection (hpi) and 24 hpi, respectively, in wild-type (WT) or *ttc21b* morphant or rescued embryos. The white arrowheads indicate the heart (scale bars, 100 μm). **(M)** Percentage fluorescent intensity remaining after 24 hpi in *ttc21b* morphants group versus WT group and WT mRNA rescued group. **(N)** A group image of 3 dpf morphology of WT embryos or embryos injected with ctrl MO, MO, or co-injected with MO and human WT *TTC21B* or mutant *TTC21B* mRNAs. Scale bars, 1000 μm. **(O)** Embryos were categorized by severity of the phenotype: the normal was categorized as no obvious change; class I indicated mild ciliopathy defects, including slight pericardial edema and/or pronephric cysts; class II referred to embryos that presented with severe ciliopathy defects, including severe pericardial edema or body curvature or hydrocephalus. Scale bars, 200 μm. **(P)** Stacked bar charts show the percentages of the normal, class I, and class II in different experimental groups. Co-injection of WT *TTC21B* mRNAs with MO resulted in significant rescue, while mutant *TTC21B* mRNAs exhibited attenuated rescue ability compared with WT mRNAs. **(Q)** Representative confocal images show whole-mount immunostaining of anti-acetylated tubulin antibody (red) labeled cilia in pronephric ducts at 27 hpf. Scale bars, 10 μm. **(R)** Histogram analysis of cilia length (measured by ImageJ software). Cilia's length was decreased in *ttc21b* morphant compared with the WT embryo. Whereas cilia defects were well rescued by co-injected with human WT *TTC21B* mRNAs, the variant *TTC21B* mRNAs failed to effectively rescue the defects. Data were represented as mean ± standard deviation. ∗*P* < 0.05, ∗∗*P* < 0.01, and ∗∗∗*P* < 0.001.

Subsequently, we generated a zebrafish model using morpholino technology. Knockdown of the *ttc21b* gene resulted in typical manifestations of ciliopathy at 3 days post-fertilization (dpf), including a curved body axis, hydrocephalus, pericardial edema, pronephric cysts, and abnormal otoliths in otic vesicles (Fig. 1H). Whole-mount *in situ* hybridization analyses of Wilms tumor gene *wt1a*, a podocyte marker, confirmed the presence of pronephric cysts (Fig. 1J). To evaluate renal function in *ttc21b* morphants, rhodamine-labeled 10-kDa dextran was injected into the pericardium of 3-dpf embryos, and dextran excretions were calculated at 24 h post-injection (hpi). In wild-type (WT) embryos, nearly all the dextran was excreted by 24 hpi, while approximately 72.1% of dextran remained in the pericardial cavity of *ttc21b* morphants, indicating impaired clearance of low molecular weight components (Fig. 1L, M). Importantly, all *ttc21b* morpholino-induced defects were significantly rescued by overexpression of human WT *TTC21B* mRNA (Fig. 1I, K, M).

Next, we performed *in vivo* complementation tests to determine the pathogenicity of the *TTC21B* missense variants. We co-injected the *ttc21b* morpholinos with each human variant mRNA (p.P209L, p.C518R, and p.C299R) into zebrafish embryos and compared the rescue efficiency with WT *TTC21B* mRNA. Phenotypic alterations were classified into three categories: appear to be normal; mild ciliopathy defects (class I), such as slight pericardial edema and/or pronephric cysts; and severe ciliopathy phenotypes (class II), including pronounced pericardial edema, body curvature, or hydrocephalus (Fig. 1O). Notably, 84.4% of *ttc21b* morphants exhibited severe ciliopathy phenotypes, which was decreased to 18.4% by co-injection with WT *TTC21B* mRNA (Fig. 1N–P). Interestingly, the rescue efficacy of all variant mRNAs was significantly attenuated (Fig. 1N–P). Furthermore, we analyzed the role of *ttc21b* in ciliary formation. The results showed that the anterior pronephric cilia length was dramatically shortened in *ttc21b* morphants at 27 h post-fertilization (hpf) compared with WT embryos. Similarly, co-injection with WT *TTC21B* mRNA resulted in a complete restoration of cilia length, while the mutant *TTC21B* mRNAs only partially restored cilia length (Fig. 1Q, R). These results underscore the pathogenicity of the p.C518R and p.C299R variants *in vivo*.

Then, we performed whole-mount *in situ* hybridization using the cardiac-specific marker cardiac myosin light chain 2 (*cmlc2*) and the liver marker succinate-CoA ligase GDP-forming subunit beta (*suclg2*) to assess the impact of the *ttc21b* gene variants on the development of left-right asymmetry. Our findings revealed that 98.6% of control morpholino-injected embryos exhibited a leftward heart tube jog compared with 34.6% of *ttc21b* morphants (Fig. S1A, B). Consistent with the *cmlc2* findings, a significant proportion of *ttc21b* morphants (42.4%) displayed a reversed *suclg2* expression pattern (Fig. S1C, D). Moreover, we collected the 18-hpf and 19.5-hpf embryos to detect the expression patterns of the Nodal-related gene *spaw* and paired-like homeodomain transcription factor 2c (*pitx2c*), respectively, both of which are critical for situs-specific organogenesis. Our analysis showed that only 30.3% of *ttc21b* morphant embryos had left-side lateral plate mesoderm *spaw* expression, compared with 75.0% in controls (Fig. S1E, F). The expression pattern of *pitx2c* alteration after *ttc21b* ablation resembled that of the *spaw* (Fig. S1G, H). Moreover, these aberrant expression patterns observed in *ttc21b morphants* were significantly ameliorated by co-injection with WT *TTC21B* mRNA (Fig. S1F, H). Collectively, these findings demonstrate that *ttc21b* is required for the left-right patterning of internal organs during zebrafish embryogenesis.

Further, to evaluate the formation of Kupffer's vesicle (KV) cilia, which is crucial for the development of left-right asymmetric organs, *ttc21b*-morpholino was injected into one- or two-cell stage Tg(sox17:gfp) embryos, followed by immunostaining with anti-GFP and anti-acetylated tubulin antibodies at the 10-somite stage. Although the number of KV cilia remained unchanged, the average cilia length was significantly shorter in *ttc21b* morphants compared with WT embryos (Fig. S2A, D, E). Subsequently, examination of KV formation in live embryos at the 10-somite stage revealed a significant impact on KV morphogenesis due to *ttc21b* knockdown (Fig. S2B, F). *Charon* (DAN domain BMP antagonist family member 5/*dand5*) is the initial gene exhibiting asymmetric expression, influenced by the intensity and direction of KV flow during early embryonic development. In WT embryos, asymmetric *charon* expression was detected on the right side of the KV. Conversely, *ttc21b* morphants exhibited a disturbed expression pattern with a higher proportion on the left side and no directional bias (Fig. S2C, G). These findings highlight the critical role of *ttc21b* in zebrafish KV formation and fluid flow regulation.

To further substantiate the findings observed in *ttc21b* morphants, we generated *ttc21b* mutants with a 3-bp deletion and a 2-bp insertion in exon 3 using CRISPR-Cas9 (Fig. S3A). At 72 hpf, all homozygous mutants exhibited kidney cysts, with some embryos also exhibiting mild pericardial edema. These phenotypes were ameliorated by co-expressing WT *TTC21B* mRNA (Fig. S3B–F). Notably, body curvature and growth retardation were observed at 30 dpf (Fig. S3H). Additionally, the homozygous *ttc21b* mutants exhibited shorter cilia in the pronephric duct compared with their WT siblings (Fig. S3I, J). However, only 7% (4/57) of the *ttc21b* mutant embryos showed a no-loop heart, and 4.3% (2/45) presented with a right-sided liver at 72 hpf (Fig. S4A–D). Regrettably, most of the homozygous *ttc21b* mutants succumbed within 30 days (Fig. S3G), and those that survived to adulthood were sterile. Consequently, homozygous embryos could only be obtained by crossing heterozygous individuals. Therefore, this discrepancy may be attributed to the presence of the maternally derived WT *ttc21b* mRNAs. Collectively, these results indicate the crucial role of *ttc21b* in ciliogenesis and the development of left-right asymmetry.

In conclusion, our study has identified 4 novel *TTC21B* variants and indicated a hotspot variant (c.1552T > C, p.C518R) that appears to be unique in Chinese patients with NPHP12. The *in vivo* studies conducted in zebrafish have confirmed the pathogenicity of *TTC21B* missense variants and demonstrated a novel association of *TTC21B* in regulating the development of left-right asymmetry.

## CRediT authorship contribution statement

**Linxia Deng:** Writing – original draft, Project administration, Methodology, Formal analysis, Data curation. **Yuan Yang:** Writing – original draft, Project administration, Data curation. **Xiaoling Yin:** Writing – original draft, Project administration, Formal analysis. **Jing Yang:** Writing – original draft, Project administration, Formal analysis. **Yijie Duan:** Formal analysis, Data curation. **Kang Wang:** Formal analysis, Data curation. **Weicheng Duan:** Data curation. **Yu Zhang:** Supervision, Conceptualization. **Bo Xiong:** Writing – review & editing, Supervision, Data curation, Conceptualization. **Jianhua Zhou:** Writing – review & editing, Supervision, Funding acquisition, Conceptualization.

## Ethics declaration

This study was approved by the Human Ethics Committees of the Tongji Hospital, Tongji Medical College, Huazhong University of Science and Technology (approval number: No.TJ-IRB20220950). Written informed consent was obtained from participants or their parents.

## Data availability

The data are available upon request from the authors.

## Funding

This work was supported by the 10.13039/501100001809National Natural Science Foundation of China (No. 81873596), the Key Research and Development Program of Hubei Province, China (No. 2022BCA047), and the 10.13039/501100012166National Key Research and Development Program of China (No. 2022YFC2705102 and No. 2022YFC2705103).

## Conflict of interests

The authors declared no competing interests.
