# Supplementary material for: TTC21B variants disrupt the left-right asymmetry and pronephric development in zebrafish
Source: Genes Dis. 2025 Jun 6;13(2):101709. doi: 10.1016/j.gendis.2025.101709 (PMC12596562; doi:10.1016/j.gendis.2025.101709)
Supplement: Multimedia component 1 [file mmc1.docx]

**Supplementary methods**

**Subjects and Clinical Data**

5 cases of NPHP12 patients from 4 non-consanguineous and their family members were included in this study. Clinical and laboratory data were obtained from existing medical records and telephone follow-ups.

**Data Source and Search Strategy**

Systematic literature searches were conducted in databases such as MEDLINE, Google Scholar, Embase, and Chinese databases including Wanfang and CNKI to identify studies related to the TTC21B gene. The search terms used were “nephronophthisis”, “TTC21B”, “IFT139”, “short-rib thoracic dysplasia 4” or “Joubert syndrome”. Following a comprehensive review of the retrieved literature, we screened for cases with *TTC21B* variants reported in the Chinese population. Subsequently, we extracted detailed clinical information from patients carrying the p.C518R mutation in the *TTC21B* gene and included them in our study cohort.

**Exome Sequencing**

Exome sequencing (ES) was performed following established protocols[1]. Briefly, genomic DNA was isolated from peripheral blood leukocytes (PBLs). The DNA was fragmented using a Covaris LE220 Sonicator (Covaris, USA) and then repaired with an end-repair mix before being ligated with adapters. The adapter-ligated DNA fragments were amplified to generate a paired-end library (150–200 bp). Exome captures were performed with Agilent SureSelect Human All Exon Kit v4 (Agilent Technologies, USA) and next-generation sequencing (NGS) was performed on the Illumina HiSeq X platform. The sequencing data were mapped to the human genome reference (hg19) utilizing Sentieon BWA software and the variants were annotated by VEP software. Variant calling and filtering were conducted by experienced geneticists.

**Zebrafish Maintenance and Staging**

All zebraﬁsh experiments were approved by the Tongji Hospital Animal Care and Use Committee. The wild-type TU line was obtained and maintained in Zebrafish Experimental Animal Centers, Tongji Medical College of Huazhong University of Science and Technology. Transgenic Tg(sox17:GFP) zebrafish were obtained from the China Zebrafish Resource Center.

**Plasmid Construction, RNA Synthesis, and Injection of Morpholino and mRNA**

The full-length coding sequences of wild-type human *TTC21B* were amplified and cloned into the pCS2^+^ plasmid. Point mutations were generated by whole plasimid PCR using the pCS2^+^+*TTC21B* plasmid as a template. All constructs were confirmed by complete sequencing. Capped mRNAs were synthesized *in vitro* using the mMESSAGE mMACHINE™ T7 Transcription Kit (Ambion, USA). The sequences of the *ttc21b* morpholinos (MO) were described in a previous study[2]. For knockdown experiments, 4 ng MO were injected into one-cell-stage wild-type or Tg(sox17:GFP) embryos to suppress *ttc21b* expression. For rescue experiments, 50pg mRNA were co-injected with *ttc21b* MO at one-cell stage.

**Renal Function Assessment**

The renal function assay was performed following established methodologies as previously described[3]. The 72-hpf wild type and *ttc21b* morphants zebrafish were anesthetized by 0.01% tricaine. Then 1 nl of 5 mg/ml 10 kDa Rhodamine-labeled dextran (Sigma-Aldrich, UK) was microinjected into the pericardial cavity, and the initial fluorescence intensity was recorded using the fluorescent stereomicroscope Axio Zoom V16 (Zeiss, Germany). 24 hours post-injection (the following day), the residual fluorescence intensity within the pericardium was quantified to assess dextran clearance by the pronephros.

**Whole-Mount In Situ Hybridizations**

Whole-mount in situ hybridization was carried out with standard protocols as previously described[4]. PCR template-derived probes for *ttc21b*, *wt1a*, *suclg2*, and *charon* were synthesized as described in prior studies[5]. Complementary DNA fragments of *spaw* and *cmlc2* were cloned into the pGM-T Fast Vector. Antisense RNA probes were labeled with digoxigenin (Roche, Basel, Switzerland) through in vitro transcription.

**Whole-mount Immunostaining and Confocal Microscope**

Whole-mount immunostaining was performed in accordance with established protocols[6, 7]. Embryos were fixed in 4% paraformaldehyde at 4℃ overnight, followed by progressive rehydration with methanol and storage at -20°C for a minimum of 48 hours. Subsequently, the fixed embryos underwent progressive rehydrated and then washed 3 times with PBTx (0.5% Triton X-100 in PBS) for 5 minutes each. The embryos were then blocked in 10% fetal calf serum in PBST at room temperature for 1 hour, followed by overnight incubation at 4°C with diluted primary antibodies: anti-α-Tubulin (1:200; T6793, Sigma-Aldrich, USA) and anti-GFP (1:200; 50430-2-AP, Proteintech). Post-incubation, the samples were washed twice with PBST for 30 minutes each and subsequently stained with secondary antibodies: Fluorescein (FITC)–conjugated Affinipure Goat Anti-Rabbit IgG(H+L) (1:200, SA00003-2, Proteintech) and CoraLite594–conjugated Goat Anti-Mouse IgG(H+L) (1:200, SA00013-3, Proteintech). Imaging of the cilia was conducted using a Zeiss LSM800 confocal microscope equipped with a 60x water-immersion objective lens, acquiring images as a Z-series with a step size of 0.5 μm. The number and length of cilia were quantified using ImageJ software.

**Generation of *ttc21b* mutants with CRISPR/Cas9**

*Ttc21b* mutants were generated using a CRISPR-Cas9 system targeting exon 6. Single guide RNAs (sgRNAs) were designed by the CHOPCHOP tool, and templates for in vitro transcription were generated by PCR with specific primers (supplemental Table 1). The mRNA of specific sgRNAs were derived from in vitro transcription using the T7 Transcript Kit (Roche). Cas9 capped mRNAs were transcribed from linearized Cas9 plasmid using the T3 mMessage mMachine mRNA transcription synthesis kits (Ambion). Subsequently, mixtures containing 50pg sgRNAs and 350pg Cas9 mRNAs were injected into 1- to 2-cell stage wild-type embryos, which were then raised to adulthood as the F0 generation. Genomic DNA was extracted from tail snips, and PCR was performed to amplify the fragment containing the sgRNA target site. Direct sequencing was then conducted to assess DNA sequence variants induced by the *ttc21b* sgRNAs. The chimeric F0 generation zebrafish were crossed with wild-type animals for two successive generations. Finally, an F3 screening was performed to identify the mutant alleles.

**Supplementary results**

**Genotype-Phenotype Correlation in 5 NPHP12 Patients**

In this study, we examined 5 cases of NPHP12 from 4 non-consanguineous families diagnosed at our hospital between 2016 and 2022. The proband (patient 1-1) from family 1 is a 5-year-and-8-month-old male who was admitted with a four-day history of abdominal pain. Upon admission, clinical evaluation revealed hypertension (146/100 mmHg), elevated liver enzymes, liver cirrhosis, portal hypertension, hyperparathyroidism, significant proteinuria (1034 mg/24 hours), and elevated serum creatinine (370 µmol/L). His estimated glomerular filtration rate (eGFR) was markedly reduced to 14.4 mL/min/1.73 m², consistent with end-stage renal disease (ESRD) (Table S1). The family history was significant for a deceased elder sister (patient 1-2), who also progressed to ESRD at a similar age (Fig 1A). Additionally, the sister exhibited situs inversus and an atrial septal defect (Fig 1E and Table S1). ES identified compound heterozygous mutations c.895 T>C (p.C299R) and c.1552T>C (p.C518R) in exons 9 and 13 of the *TTC21B* gene, respectively (Fig 1B). The paternally inherited missense variant, p.C299R, has been documented in the gnomAD database with a frequency of 0.000006857 (11 out of 1,604,274), with the majority (8 out of 11) of these alleles identified in individuals of East Asian descent. This substitution at residue 299 is predicted to be damaging and deleterious according to analyses by PolyPhen-2 and SIFT, and it has been classified as a variant of uncertain significance (VUS) in accordance with ACMG guidelines. Another missense variant, p.C518R, exhibits an allele frequency of 6.196e-7 (1 out of 1,613,996) and has similarly been detected exclusively in East Asians (0.00002228, 1 out of 44,874) within the gnomAD database. This variant is also predicted to be probably damaging and deleterious by PolyPhen-2 and SIFT and is classified as a VUS by ACMG guidelines. Regrettably, the genetic information for patient 1-2 is unavailable. It is hypothesized that she carries the same gene mutations as her brother (patient 1-1), as both progressed to ESRD at the same age.

Interestingly, both patient 2-1 and patient 3-1 possessed the same heterozygous missense variant, c.1552T>C (p.C518R), alongside distinct novel nonsense variants: c.1897C>T, p.Q633* and c.2581C>T, p.Q861*, respectively (Fig 1A and 1C). These nonsense variants, which are absent from public databases, were predicted through bioinformatics analyses to generate premature termination codons (PTCs), leading to frameshift-mediated protein truncation. According to the ACMG guidelines, both variants were classified as pathogenic, likely impairing gene function through mechanisms of haploinsufficiency or dominant-negative effects. Patient 2-1, a 1.5-year-old female, was admitted to our hospital with a six-month history of failure to thrive and a two-day history of poor appetite. Upon admission, she was diagnosed with hypertension (103/56 mmHg) and exhibited significant growth retardation in both weight and length. Laboratory evaluations indicated proteinuria (290.7 mg/24h), impaired renal function (serum creatinine: 105 µmol/L, eGFR: 30.7 mL/min/1.73 m²), hyperparathyroidism, and situs inversus (Table S1, Fig 1F). By the age of two, the patient progressed to ESRD and subsequently underwent a successful renal transplantation. Patient 3-1 is a male child aged 2 years and 11 months who was admitted to the hospital presenting with symptoms including cough, lethargy, and dyspnea. Upon clinical evaluation at the time of admission, the patient exhibited hypertension (130/99 mmHg), oliguria (100-200 mL/day), proteinuria, and ESRD, as indicated by a serum creatinine level of 323.9 µmol/L and eGFR of 13.8 mL/min/1.73 m². Additionally, the patient was diagnosed with situs inversus (refer to Table S1 and Fig. 1G). Two months subsequent to admission, the patient successfully underwent renal transplantation.

In family 4, the patient was a 9-year-old boy who presented with enuresis, nocturia, impaired renal function, moderate proteinuria, hypertension, and tricuspid and mitral valve regurgitation (refer to Table S1). Within six months, this patient progressed to ESRD. ES data indicated the presence of biallelic variants c.215_216delGT (p.C72Ffs*10) and c.262+5G>C in the *TTC21B* gene (refer to Fig. 1A and 1D). These variants have not been documented in any public databases. The frameshift deletion mutation is predicted to lead to truncated or nonfunctional proteins and has been classified as a likely pathogenic variant, whereas the splicing mutation c.262+5G>C has been classified as a VUS.

**Supplementary figures**

**
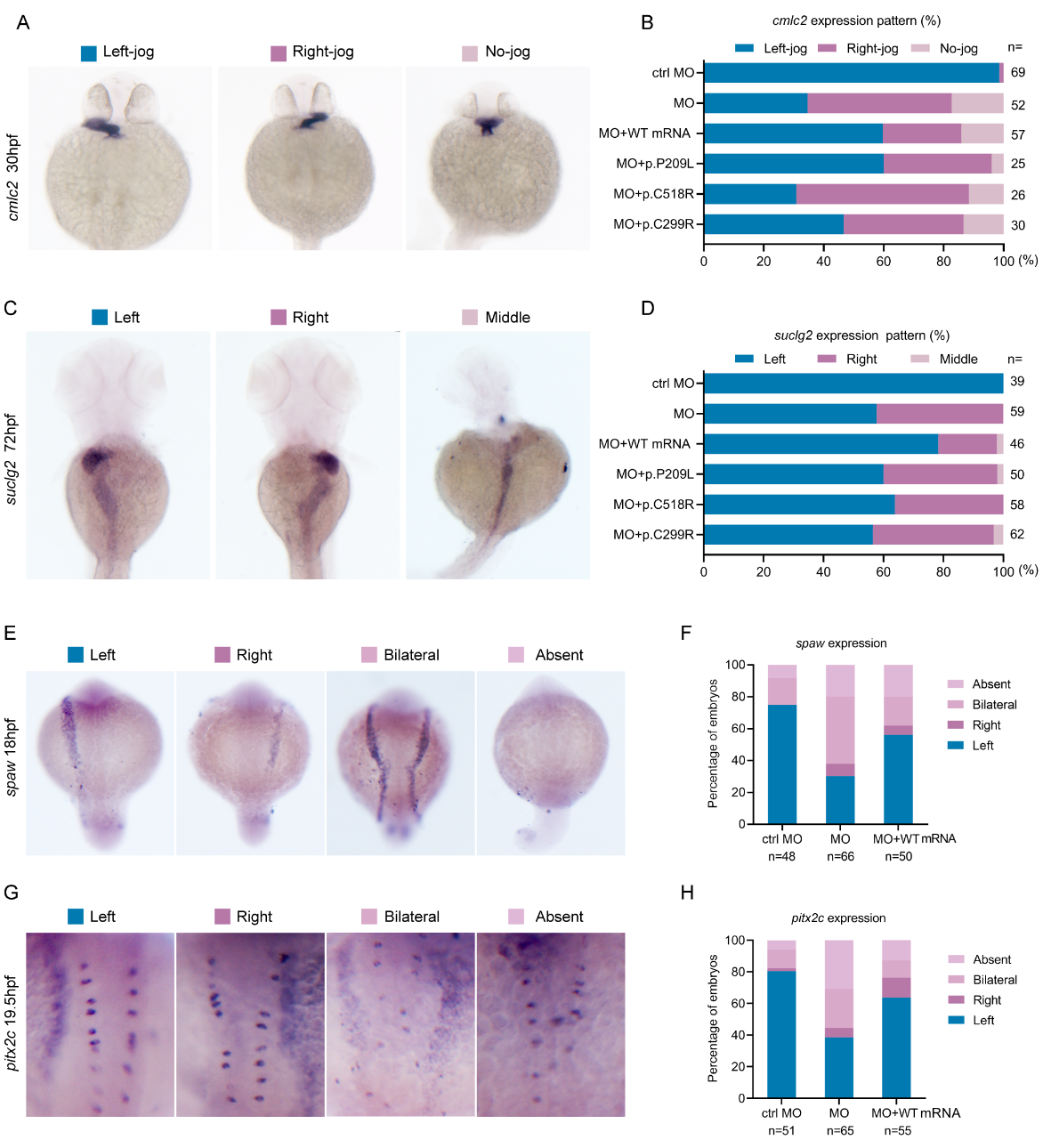
**

**Fig. S1: Suppression of *ttc21b* leads to defects in left-right asymmetry during zebraﬁsh embryogenesis.** Embryos were injected at the 1-to 2-cell stage with control MO (ctrl MO) or *ttc21b* MO (MO), with or without *TTC21B* mRNA. Cardiac jog, liver laterality, and nodal-pitx2c expression were assessed using whole-mount in situ hybridization with probes cmlc2(A), suclg2(C), spaw(E), and pitx2c(G). Embryos phenotypes are shown in top dorsal view (A) and dorsal view (C, E, G). B, D, F, H display stacked bar plots showing the percentage of different expression patterns. n, number of observed embryos.

**
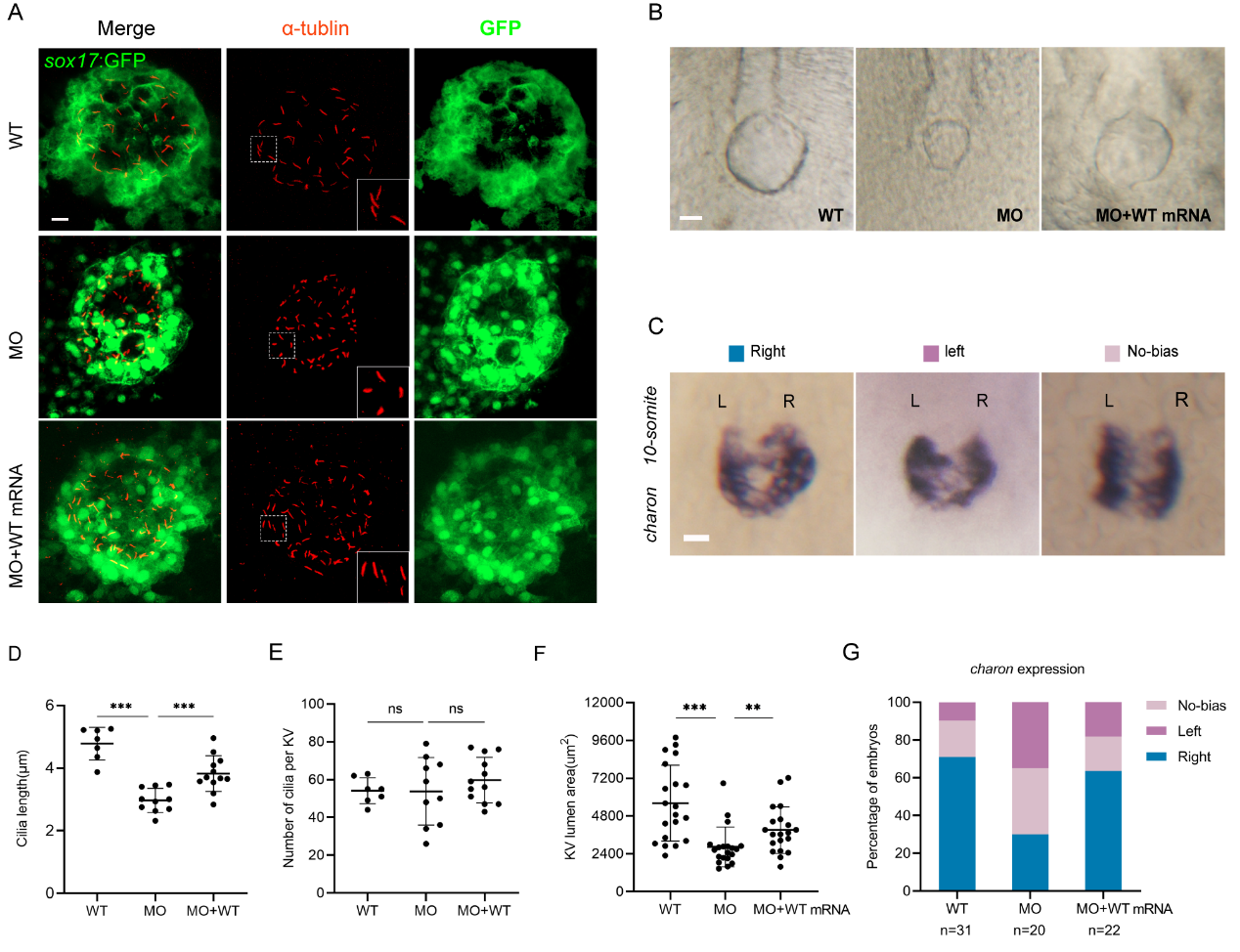
**

**Fig. S2: Depletion of *ttc21b* impaired KV cilia formation, KV lumen, and *charon* expression pattern in KV.** (A) Representative immunofluorescence images of KVs stained with anti-GFP and anti-α-Tubulin antibodies at the 10-somites. Insets are higher-power images of the boxed areas. Scale bar, 10 μm. (B) Live light micrographs image of KV morphology at 10-somites. (C) and (G) show the expression pattern of *charon* in KV and the ratio of different patterns at the 10-somites stage. (D) and (E) show the average KV cilia length and average KV cilia number per KV, respectively. The group values are expressed as the mean ±SD. ***, P <0.001 and **, P <0. 01 in Student’s t-test. Scale bar, 20μm. (F) KV lumen area analyzed with Image J, ***, P <0.01(Student’s t-test).

**
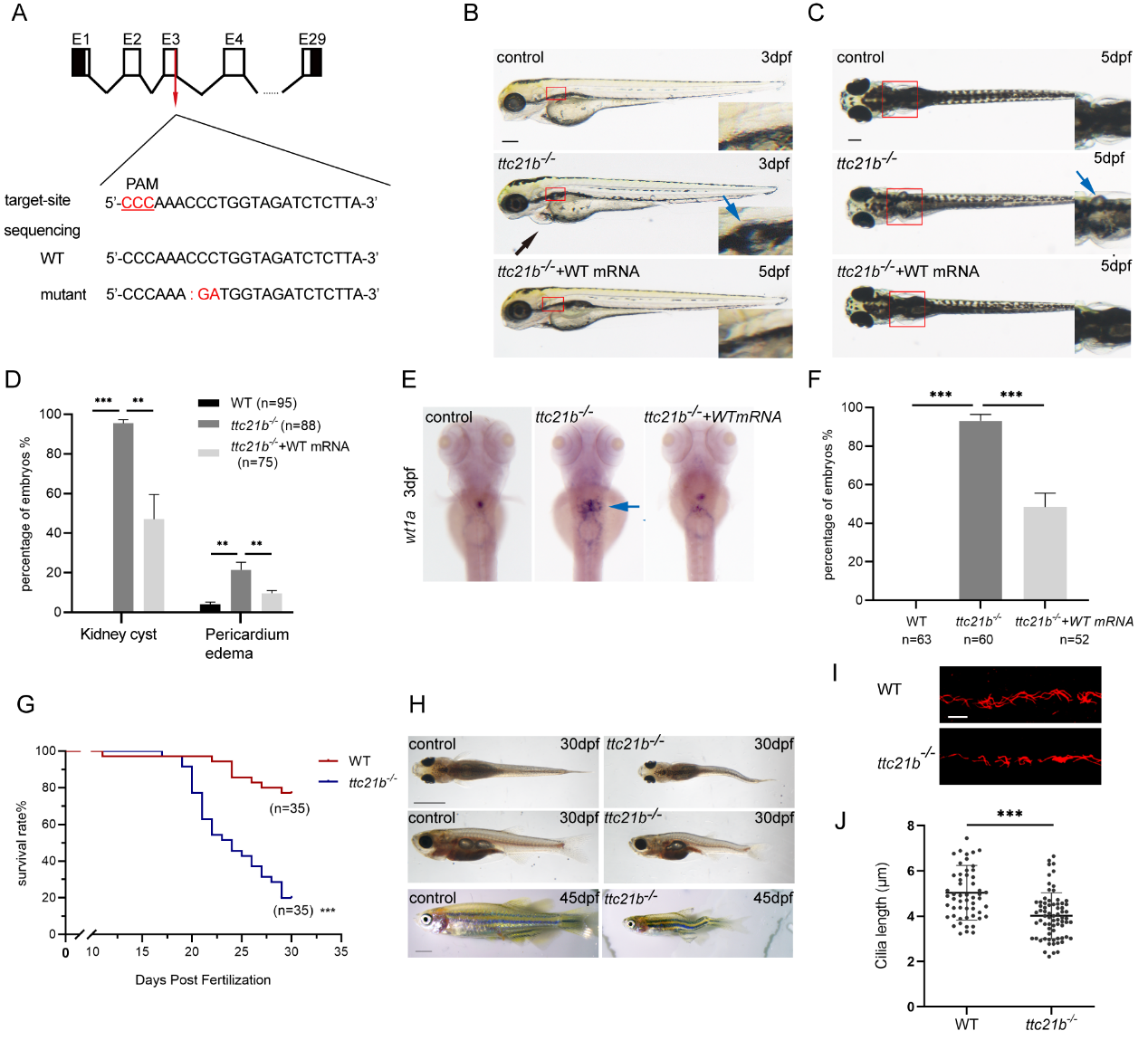
**

**Fig. S3: *ttc21b* mutants generated by the CRISPR/Cas9 system show cilia defects.** (A) The schematic shows the single guide RNA target site and the induced indel mutation in *ttc21b* mutant zebrafish. (B) and (C)*ttc21b* mutants display kidney cysts (blue arrow) and slight pericardial edema (black arrow) at 3dpf and 5 dpf respectively. Scale bar, 200μm. (D) Quantification of embryos with kidney cyst and pericardium edema. (E) Pronephric cysts validated by wholemount in situ hybridization with the podocyte differentiation marker *wt1a*. (F) Percentage of embryos with pronephric cysts. (G) Kaplan-Meier survival curves for *ttc21b* mutants vs. wild-type zebrafish, with a log-rank test showing ***, p < 0.001. (H) *ttc21b* mutants exhibit a curved body at 30dpf and 45dpf. Abnormal swim bladders were observed at 30dpf (red arrow). Scale bar, 2mm. (I) and (J) show cilia defects in the anterior pronephros of *ttc21b* mutants at 27hpf. Scale bar, 10μm.


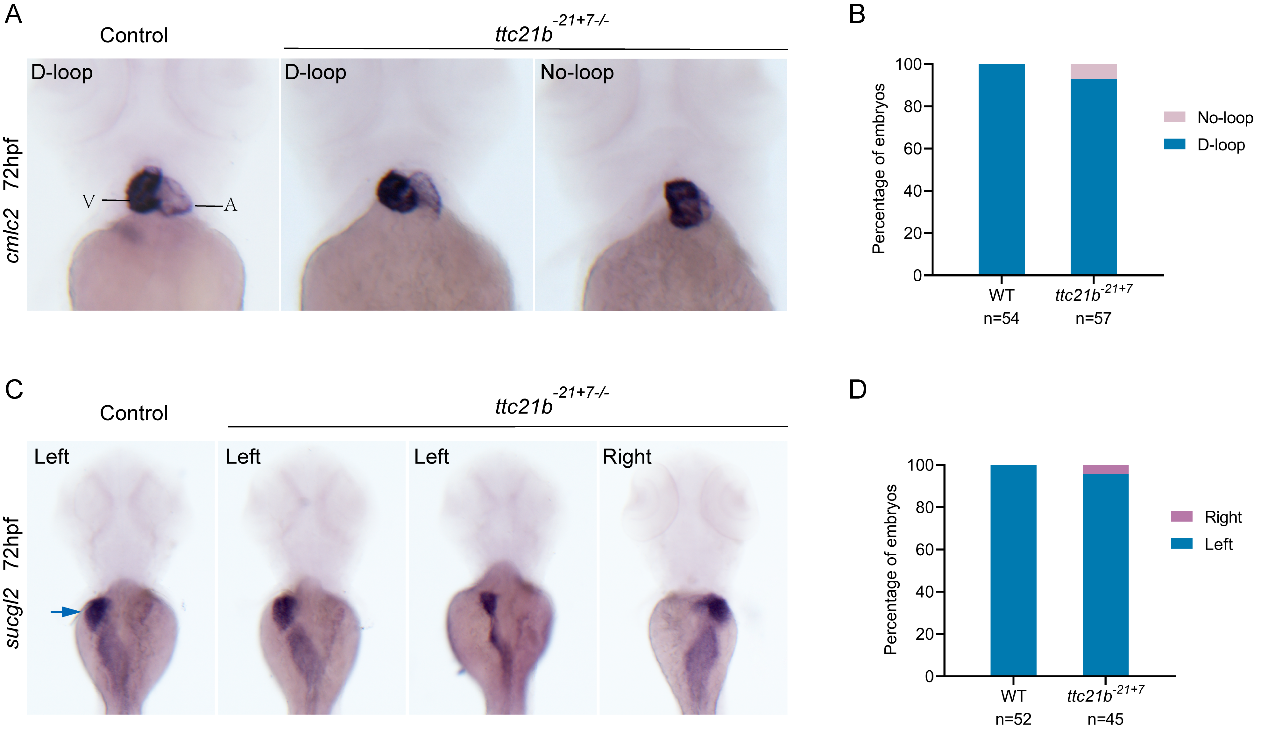


**Fig. S4: Development of left-right asymmetry in *ttc21b* mutants.** (A) Wild-type (WT) and *ttc21b* mutants stained with *cmlc2* displayed D-loop or No-loop at 72hpf. (C) WT or *ttc21b* mutants stained with *suclg2*, a liver marker, revealed left-side liver or right-side liver. (B) and (D) Ratios of embryos with different expression patterns of *cmlc2* and *suclg2* at 72hpf in WT embryos or *ttc21b* mutants.

**Supplementary tables**

**Table S1.** **Clinical features of patients with p.C518R mutation in *TTC21B* gene.**

| ID | Onset age | Age of ESRD | Gender | Variant 1 | Variant 2 | Hypertension | Proteinuria | sCr (μmol/L) | eGFR(mL/min  /1.73 m^2^) | Extra-renal phenotypes | Clinical outcome | Ref |
| --- | --- | --- | --- | --- | --- | --- | --- | --- | --- | --- | --- | --- |
| patient 1-1 | NA | 5y8m | M | c.1552T>C (p.C518R) | c.895T>C  (p.C299R) | + | + | 370 | 14.4 | Liver cirrhosis; splenomegaly | Hemodialysis |  |
| Patient 1-2 | NA | 5y7m | F | NA | NA | + | + | 643.8 | 8.3 | situs inversus;  atrial septal defect | Died |  |
| Patient 2-1 | 1y6m | 2y | F | c.1552T>C (p.C518R) | c.1897C>T  (p.Q633*) | + | + | 105 | 30.7 | Situs inversus | Kidney transplant |  |
| Patient 3-1 | NA | 2y11m | M | c.1552T>C (p.C518R) | c.2581C>T  (p.Q861*) | + | + | 323.9 | 13.8 | Situs inversus;  atrial septal defects; hepatomegaly | Kidney transplant |  |
| Patient 4-1 | 10m | 1y | F | c.1552T>C (p.C518R) | c.1456dupA (p.R486Kfs*22) | + | + | 78.8 | NA | Hepatomegaly; splenomegaly | Died | [8] |
| Patient 5-1 | 3y6m | 3y11m | F | c.1552T>C (p.C518R) | c.752T>G  (p.M251R) | + | + | 462 | 8.1 | Situs inversus; brachy-dactyl;  mitral insufficiency | NA | [9] |
| Patient 6-1 | NA | 16y | F | c.1552T>C (p.C518R) | c.2309A>G  (p.H770R) | + | + | 665.1 | NA | － | Peritoneal dialysis | [10] |
| Patient 7-1 | NA | NA | M | c.1552T>C (p.C518R) | c.1656_1659del  (p.C552fs) | + | NA | NA | NA | Situs inversus | NA | [11] |

NA: Not available or not mentioned; F: female; M: male; sCr: serum Creatinine; eGFR: estimated glomerular filtration rate; PFO: patent foramen ovale; PDA: patent ductus arteriosus; Ref: Reference.

**Table S1. Clinical features of patients with p.C518R mutation in *TTC21B* gene.**

| ID | Onset age | Age of ESRD | Gender | Variant 1 | Variant 2 | Hypertension | Proteinuria | sCr (μmol/L) | eGFR(mL/min  /1.73 m^2^) | Extra-renal phenotypes | Clinical outcome | Ref |
| --- | --- | --- | --- | --- | --- | --- | --- | --- | --- | --- | --- | --- |
| Patient 7-2 | 2m | NA | F | c.1552T>C  (p.C518R) | c.1656_1659del  (p.C552fs) | － | NA | NA | NA | Neonatal cholestasis | NA | [11] |
| Patient 8-1 | NA | 12d | F | c.497delA  (p.K166fs*36) | c.1552T>C  (p.C518R) | NA | + | 386 | 4.73 | Irregular ribs; Situs inversus; PFO and PDA | Died | [12] |
| Patient 9-1 | NA | 3y2m | F | c.1552T>C  (P.C 518R) | C.1231C>T  (p.R411X) | ＋ | ＋ | 606 | NA | Situs inversus | Peritoneal dialysis | [13] |
| Patient 9-2 | NA | 2y11m | M | c.1552T>C  (P.C 518R) | C.1231C>T  (p.R411X) | ＋ | + | 102 | NA | Hepatomegaly | Peritoneal dialysis | [13] |
| Patient 10-1 | NA | 3y11m | M | c.1552T>C  (P.C 518R) | c.1675‐1G>T | ＋ | ＋ | 271 | NA | Dextrocardia | Died | [13] |
| patient 10-2 | NA | 6m | F | c.1552T>C  (P.C 518R) | c.1675‐1G>T | － | ＋ | 88 | NA | － | Died | [13] |
| Patient 11-1 | NA | 1y3m | F | c.1552T>C  (P.C 518R) | c.1327C>G  (p.L443V) | ＋ | ＋ | 165 | NA | － | Peritoneal dialysis | [13] |
| Patient 12-1 | NA | 2y1m | M | c.1552T>C  (P.C518R) | c.530delA  (p.D177fs) | ＋ | ＋ | 158 | NA | － | Peritoneal dialysis | [13] |
| Patient 13-1 | NA | NA | M | c.1552T>C  (P.C518R) | c.1656_1659del  (p.C552fs) | NA | ＋ | NA | NA | － | Peritoneal dialysis | [14] |
| Patient 14-1 | 1y3m | NA | F | c.1552T>C  (P.C518R) | c.497delA  (p.K166Sfs*36) | NA | ＋ | 156 | NA | Polydactyly;  Situs inversus | Symptomatic treatment | [15] |

NA: Not available or not mentioned; F: female; M: male; sCr: serum Creatinine; eGFR: estimated glomerular filtration rate; PFO: patent foramen ovale; PDA: patent ductus arteriosus; Ref: Reference.

**Table S2. Sequences of the primers.**

| Primer name | sequence (5'→3') |
| --- | --- |
| ttc21b_CP6_F | taatacgactcactatagggCCAGAGCCGGTAGAAAGGACgttttagagctagaaatagc |
| ZF_cp_R | AGCACCGACTCGGTGCCACT |
| zIFT139-WISH-F | CGGAAGCAGGACTATGAACAGG |
| zIFT139-WISH-R | TAATACGACTCACTATAGGGCATGATTGAGCACCAGCACAC |
| cmlc2-F | TGGGTGTCCATGTAGGGGA |
| cmlc2-R | taatacgactcactatagggTTCCAGCCACGTCTATTGGAG |
| suclg2-F | TTGTCAGGCGGCAGCGAG |
| suclg2-R | taatacgactcactatagggGCAAGGCCAGCACCATTA |
| spaw-F | TTACAGAACTCCTACAGCAATGG |
| spaw-R | taatacgactcactatagggCATCAATGACAGCCGCACTC |
| pitx2c-F | TCACTCTGGCTCTCCTTGCT |
| pitx2c-R | taatacgactcactatagggACATGTCATCGTAGGGCTGC |
| Charon-F | CCGTTAGTCATGTGCCGTTCAG |
| Charon-R | taatacgactcactatagggACGTTTCTGTTTGCAGGGACTC |
| wt1a-wish-F | TGGCTGTCACACTCCTTCTG |
| wt1a-wish-R | taatacgactcactatagggTTGGAGCTTGTTCCTCTAGGTG |
| Zttc21b-qpcr-F | AGGGAATTGTGCTAAAAGGC |
| Zttc21b-qpcr-R | TCCCCATCAGAGCAAAAATG |
| β-actin-F | CGTGCTGTCTTCCCATCCA |
| β-actin-R | TCACCAACGTAGCTGTCTTTCTG |
| ttc21b-sp-F | CAGGATCTGGATGGACGGGT |
| ttc21b-sp-R | TGAACCTTCCCCATCAGAGCA |
| P.C518R-F | GCTTTCAATAACCTTCAGCACCGCTTAGAACACAATCCCTC |
| P.C518R-R | GAGGGATTGTGTTCTAAGCGGTGCTGAAGGTTATTGAAAGC |
| p.C299R-F | CTCGCCTTCAGCAGAACTCGTGGACGTAGTCAACTTATTC |
| p.C299R-R | GAATAAGTTGACTACGTCCACGAGTTCTGCTGAAGGCGAG |
| p.P209L-F | GAACCAGATAATCGTGAATTTTCTGAGCTTCCTTCCTGCTTT |
| p.P209L-R | AAAGCAGGAAGGAAGCTCAGAAAATTCACGATTATCTGGTTC |

**Supplementary references**

1. Deng, L.-X., et al., *A Presumed Synonymous Mutation of PKD2 Caused Autosomal Dominant Polycystic Kidney Disease in a Chinese Family.* Current Medical Science, 2021. **41**(5): p. 1029-1036.

2. Davis, E.E., et al., *TTC21B contributes both causal and modifying alleles across the ciliopathy spectrum.* Nat Genet, 2011. **43**(3): p. 189-96.

3. Christou-Savina, S., P.L. Beales, and D.P. Osborn, *Evaluation of zebrafish kidney function using a fluorescent clearance assay.* J Vis Exp, 2015(96): p. e52540.

4. Thisse, C. and B. Thisse, *High-resolution in situ hybridization to whole-mount zebrafish embryos.* Nat Protoc, 2008. **3**(1): p. 59-69.

5. Cha, Y.R. and B.M. Weinstein, *Use of PCR template-derived probes prevents off-target whole mount in situ hybridization in transgenic zebrafish.* Zebrafish, 2012. **9**(2): p. 85-9.

6. Rothschild, S.C., L. Francescatto, and R.M. Tombes, *Immunostaining Phospho-epitopes in Ciliated Organs of Whole Mount Zebrafish Embryos.* J Vis Exp, 2016(108): p. 53747.

7. Molinari, E., et al., *Using zebrafish to study the function of nephronophthisis and related ciliopathy genes.* F1000Research, 2018. **7**: p. 1133.

8. Zhang, H., et al., *Mutations in TTC21B cause different phenotypes in two childhood cases in China.* Nephrology, 2018. **23**(4): p. 371-376.

9. Jian, S., et al., *Clinical features and TTC21B genotype of a child with nephronophthisis type 12.* Zhongguo Dang Dai Er Ke Za Zhi, 2019. **21**(6): p. 580-584.

10. Liu, L., et al., *A case of proliferative glomerulosclerosis with compound heterozygous TTC21B mutations.* Clin Chim Acta, 2022. **529**: p. 17-20.

11. Chen, W., et al., *Biallelic mutations of TTC12 and TTC21B were identified in Chinese patients with multisystem ciliopathy syndromes.* Human Genomics, 2022. **16**(1): p. 48.

12. Li, Y., et al., *Clinical report and genetic analysis of rare premature infant nephronophthisis caused by biallelic TTC21B variants.* Mol Genet Genomic Med, 2024. **12**(3): p. e2399.

13. Zhang, J., et al., *Clinical phenotype analysis of 6 cases of TTC21B gene related nephronophthisis.* Zhonghua Er Ke Za Zhi, 2022. **60**(8): p. 820-824.

14. Shaohan Fang, et al., *Analysis of clinical characteristics and genetic variants in 10 children with NPHP.* Anhui Medical Journal, 2021. **42**(5): p. 498-501.

15. Zhao, X., et al., *Clinical phenotype characteristics and genetic analysis in children with nephronophthisis and related syndromes caused by different gene mutations.* Zhongguo Dang Dai Er Ke Za Zhi, 2023. **25**(8): p. 831-836.
